# The Impact of Steatosis on Chronic Hepatitis C Progression and Response to Antiviral Treatments

**DOI:** 10.3390/biomedicines9101491

**Published:** 2021-10-17

**Authors:** Phumelele Yvonne Siphepho, Yi-Ting Liu, Ciniso Sylvester Shabangu, Jee-Fu Huang, Chung-Feng Huang, Ming-Lun Yeh, Ming-Lung Yu, Shu-Chi Wang

**Affiliations:** 1 Program in Tropical Medicine, Graduate Institute of Medicine, Kaohsiung Medical University, Kaohsiung 80708, Taiwan; yolliesiphepho@gmail.com (P.Y.S.); fish6069@gmail.com (M.-L.Y.); 2 Center for Cancer Research, Center for Liquid Biopsy, Kaohsiung Medical University, Kaohsiung 80708, Taiwan; u107567006@kmu.edu.tw (C.S.S.); jfliver@kmu.edu.tw (J.-F.H.); 3 Department of Medical Laboratory Science and Biotechnology, Kaohsiung Medical University, Kaohsiung 80708, Taiwan; q0987590988@gmail.com; 4 Graduate Institute of Medicine, Kaohsiung Medical University, Kaohsiung 80708, Taiwan; 5 Hepatobiliary Division, Department of Internal Medicine, Kaohsiung Medical University Hospital, Kaohsiung Medical University, Kaohsiung 80708, Taiwan; fengcheerup@gmail.com.tw (C.-F.H.); min-glunyeh@gmail.com (M.-L.Y.); 6 Faculty of Internal Medicine, School of Medicine, College of Medicine, Kaohsiung Medical University, Kaohsiung 80708, Taiwan; 7 Hepatitis Research Center, Kaohsiung Medical University, Kaohsiung 80708, Taiwan; 8 Department of Medical Research, Kaohsiung Medical University Hospital, Kaohsiung 80708, Taiwan

**Keywords:** hepatitis C virus, steatosis, NAFLD/NASH (MAFLD), metabolic syndrome, hepatocellular carcinoma, interferon-based therapy, interferon-free direct-acting antivirals

## Abstract

Metabolic derangement is characteristic in patients with hepatitis C virus (HCV) infection. Aside from established liver injury, various extrahepatic metabolic disorders impact the natural history of the disease, clinical outcomes, and the efficacy of antiviral therapy. The presence of steatosis, recently redefined as metabolic-associated fatty liver disease (MAFLD), is a common feature in HCV-infected patients, induced by host and/or viral factors. Most chronic HCV-infected (CHC) patients have mild steatosis within the periportal region of the liver with an estimated prevalence of 40% to 86%. Indeed, this is higher than the 19% to 50% prevalence observed in patients with other chronic liver diseases such as chronic hepatitis B (CHB). The histological manifestations of HCV infection are frequently observed in genotype 3 (G-3), where relative to other genotypes, the prevalence and severity of steatosis is also increased. Steatosis may independently influence the treatment efficacy of either interferon-based or interferon-free antiviral regimens. This review aimed to provide updated evidence of the prevalence and risk factors behind HCV-associated steatosis, as well as explore the impact of steatosis on HCV-related outcomes.

## 1. Introduction

Hepatic steatosis refers to the accumulation of intrahepatic fat of at least 5% of liver weight. In most cases, triacylglycerol accumulation in the liver is considered hepatoprotective; however, long-term storage of lipids could potentially cause liver metabolic dysfunction, as well as steatosis including nonalcoholic fatty liver disease (NAFLD) and its more advanced clinical manifestation, nonalcoholic steatohepatitis (NASH) [1]. Recently, international experts suggested a change of nomenclature from NAFLD to metabolic-associated fatty liver disease (MAFLD) to better emphasize the strong pathophysiological link between steatosis and metabolic dysfunction [2,3]; however, this suggestion is still under consideration, and the proposed criteria for MAFLD diagnosis require more research [4]. NAFLD presents in approximately 25% of the most common liver disorders worldwide, ranging from 13% in Africa to 42% in southeast Asia [5,6,7]. Even though NASH is more likely to advance to liver cirrhosis and hepatocellular carcinoma than simple steatosis, both disorders may ultimately lead to advanced fibrosis; however, this process occurs more gradually in simple steatosis [8,9,10]. NAFLD is primarily associated with metabolic syndrome (MetS), which refers to the co-occurrence of several known risk factors of cardiovascular disease (CVD) and type 2 diabetes (T2D), such as insulin resistance (IR), obesity, atherogenic dyslipidemia, and hypertension [11,12]. These risk factors have been further identified as underlying risk factors for NAFLD and NASH in 63.6% of hepatitis C virus (HCV)-related cirrhotic livers [13].

Genetic studies related to NASH risk factors found that Asians are most susceptible to MetS and NAFLD, while Hispanics have a higher prevalence than whites, and Blacks naturally have the lowest [14,15,16]. Singal et al. further suggested that the enzyme patatin-like phospholipase domain-containing 3 (PNPLA3) is linked to an increased risk of advanced fibrosis and is an independent risk factor for hepatocellular carcinoma (HCC) among patients with liver disease and NASH or alcohol-related cirrhosis, respectively [17,18]. Regarding gender, higher levels of estrogen in women have been known to increase their vulnerability to NAFLD, where the risk of glucose intolerance, insulin resistance, hyperlipidemia, and visceral fat accumulation is increased in postmenopausal women [19,20]. Since the recruitment of lipid droplets by HCV proteins is critical for viral replication, liver steatosis is frequently observed in the livers of HCV-infected patients with a prevalence of 40% to 86% [21]. This is considered higher than in patients with other chronic liver diseases (19–50%) such as chronic hepatitis B (CHB) infection [22].

The mechanism via which HCV induces steatosis is complex and includes elements involved in lipogenesis and mitochondrial β-oxidation. In HCV, viral and host factors are implicated in steatosis; hence, two types of steatosis have been defined: “viral steatosis” induced by viral proteins and “metabolic steatosis” induced by host/metabolic factors [21]. The etiology and response rates to antiviral treatments vary among the two types and further correlates with specific genotypes of HCV. In genotypes 1 and 2 of chronic hepatitis C (G-1/2 CHC)-infected patients, steatosis is mostly associated with host factors (obesity, diabetes, hypertension, and metabolic syndrome), where the severity of fat accumulation correlates to the body mass index (BMI) and degree of visceral fat. On the other hand, steatosis appears to be perpetuated by viral proteins in genotype 3 of chronic hepatitis C (G-3 CHC)-infected patients, where the degree of fat accumulation is proportional to the level of HCV replication and viral protein expression [23,24].

The two groups of HCV genotypes also differ in their clearance of steatosis following a sustained virologic response (SVR) to either interferon (IFN)-based or interferon-free direct-acting antiviral (IFN-free DAA) treatments. Contrary to metabolic steatosis in G-1/2 CHC patients, an SVR achieved with interferons significantly resolves viral steatosis in G-3 CHC patients, while HCV-related metabolic steatosis has been linked to IR, thus significantly reducing the treatment efficacy of interferon-based therapy [25]. A similar negative response to DAA treatment was observed in G-1 CHC-infected patients, where an increase in controlled attenuation parameter (CAP), a marker of liver steatosis, was reported following viral clearance [26,27,28].

Understanding the role of steatosis toward the progression of CHC-related diseases could significantly improve current treatments of HCV-related chronic liver disease. In view of the accumulating knowledge about risk factors and pathogenic features of NAFLD/NASH, further investigation of the potential ramifications of steatosis in CHC infection is imperative; accordingly, this paper was aimed at providing an updated review of the prevalence and risk factors behind steatosis in CHC infections, as well as explore the impact of steatosis on the disease course and treatment outcomes of CHC.

## 2. The Impact of HCV-Associated Steatosis on Necroinflammation, Fibrosis, and HCC

The rate of disease progression in CHC patients has been linked to several factors including age, sex, and alcohol abuse; however, it remains elusive as to whether or not steatosis should be considered a factor. Since steatosis is more persistent in patients with CHC (55%) as opposed to the remaining Western population (20–30%) [29], steatosis in HCV patients has since been implicated in major hepatocellular injury including severe fibrosis in various studies [30,31]. A study that included medical records of 603 African Americans with CHC infection at Howard University Hospital evaluated risk factors associated with the progression to liver fibrosis [32], where steatosis grade (OR 1.6, *p* = 0.002) was independently associated with fibrosis stage (3–4 vs. 0–2). A similar significant correlation was observed between the grade of steatosis and fibrosis in 180 liver biopsies derived from CHC patients [30]. In obese patients with NASH, another study discovered that a high degree of steatosis and inflammation were amongst the recognized risk factors for fibrosis [33].

While more of this correlation between steatosis and fibrosis has been reported in HCV infections [34,35], there have been contradictory findings where liver fibrosis progression was associated with other risk factors including older age, higher BMI, periportal necroinflammation, and ALT and serum ALT elevations, and less so with steatosis [36,37]. On the other hand, some studies have hinted at a higher probability of fibrosis progression in G-3 [30,38]. This G-3-specific progression in fibrosis could be justified by the aggressive nature of steatosis in this specific genotype, [39,40]. A definitive conclusion regarding this matter would require further investigation. Even though factors behind fibrosis progression in CHC are poorly understood [41], IR could potentially be the link [31,42]. Recently, a study showed that, in HCV, the presence and degree of IR correlate with the fibrosis stage, whereby IR has been known to perpetuate the fibroinflammatory process. This may be explained by the expression of proinflammatory and fibrogenic cytokines, as well as circulating adipokines, which collectively affect both insulin sensitivity and inflammation, [43,44,45]. The precise mechanisms behind these findings remain a mystery; however, the mechanisms via which steatosis and IR differently influence progression of liver fibrosis have been established, whereby IR manifests as an overproduction of hepatic glucose, while steatosis is more linked to hepatic inflammation [46].

The association between steatosis and the risk for HCC in CHC has been explored in numerous studies, as summarized in Table 1.

A study demonstrated that HCC development in CHC-infected patients following a SVR depended on age (≥55) (*p* = 0.021), hepatic fibrosis (F3–4) (*p* = 0.0028), and hepatic steatosis (grade 2–3) (*p* = 0.0002) at pre-IFN treatment, where HCC developed within a period of 10 years. Subsequently, CHC-infected patients with these underlying risk factors require constant monitoring for HCC development [48]. Similarly, in another study, hepatic steatosis was among identified risk factors for HCV-associated HCC where the tumor recurrence rate was significantly higher in steatosis-positive patients than in steatosis-negative patients (*p* = 0.02), [50]. While other studies have reported similar findings, [47,49,51,52], a genotype-specific risk for HCC has been speculated, where the risk was particularly greater in G-3 CHC-infected patients. Accordingly, a substantial proportion of patients with this genotype may potentially be at risk of CHC progression to cirrhosis and HCC, suggesting that HCV genotype status may be useful as a risk screener [53,54,55].

In as much as G-3 CHC has been linked to an increased risk for HCC, it cannot be absolutely assumed that viral steatosis is the primary connection; furthermore, this possibility was ruled out by a study which suggested an absence of steatosis in late stages of liver disease, which is at the time HCC occurs [38]. In conclusion, the relationship between HCV and steatosis regarding hepatic complications is largely speculative; however, accumulating evidence suggests that steatosis, as well as IR, might contribute to progression of fibrosis or liver disease, thereby altering the natural history of CHC. Moreover, according to the data reported in Table 1, considering the increased risk for HCC posed by steatosis, additional surveillance is necessary in HCV steatosis-positive patients, particularly those with G-3 [53]. Finally, liver steatosis may be considered a risk factor for inflammation, increased hepatic fibrosis, and liver damage or HCC in CHC patients.

## 3. Molecular Mechanisms of HCV-Associated Steatosis

**Figure 1:**
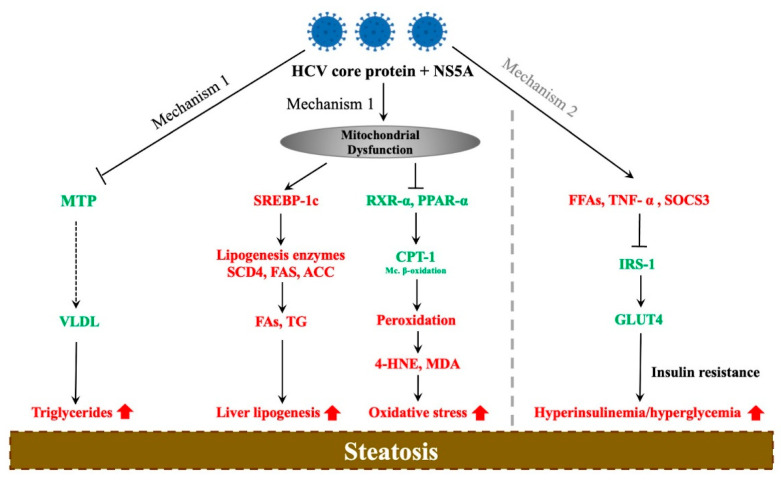
The effect of hepatitis C virus on liver steatosis development. Red and green illustrate upregulation and downregulation, respectively. HCV: hepatitis C virus; MTP: microsomal triglyceride transfer protein; VLDL: very-low-density lipoprotein; SREBP- 1c: sterol regulatory element-binding-protein 1c; SCD4: sterol CoA dehydrogenase 4; FAS: fatty-acid synthase; ACC: acetyl-CoA carboxylase; PPAR-α: peroxisome proliferator-activated receptor alpha; CPT-1: carnitine palmitoyltransferase-1; 4-HNE: 4-hydroxynonenal; MDA: malondialdehyde; FFAs: free fatty acids; TNF-α: tumor necrosis factor alpha; SOCS3: suppressor of cytokine signaling 3; IRS-1: insulin receptor substrate signaling; Mc. β-oxidation: mitochondrial β-oxidation; GLUT-4: glucose transporter type 4.

Various studies have shown that an excess of lipids in the liver is critical in maintaining the HCV life cycle. According to several pieces of evidence, HCV might directly cause lipid accumulation in hepatocytes. Firstly, steatosis is more frequent and severe in G-3 patients, suggesting the direct involvement of HCV viral proteins in the accumulation of triglycerides in hepatocytes. Secondly, observed within this very same genotype is a correlation between the degree of steatosis and level of HCV replication in the liver. Lastly, the response to antivirals among G-3 CHC patients is a major factor, where a significant reduction in fatty liver has been reported following successful treatment [24,31,39,40]. Consequently, several mechanisms have since been linked to the development of HCV-associated steatosis, illustrated in Figure 1.

The first mechanism highlights the role of the HCV core protein and NS5A in interfering with lipid metabolism. These proteins inhibit the microsomal triglyceride transfer protein (MTP), an enzyme responsible for the assembly of very-low-density lipoprotein (VLDL), thus resulting in the accumulation of triglycerides in hepatocytes leading to steatosis [56]. Mitochondrial dysfunction has also been linked to steatosis in HCV patients either through activation of the sterol regulatory element-binding-protein (SREBP 1c) signaling pathway or inhibition of retinoid X receptor alpha (RXR-α) and peroxisome proliferator-activated receptor alpha (PPAR-α); the key regulators of fatty-acid beta-oxidation. The HCV core protein upregulates SREBP-1c, thereby activating the enzymes sterol CoA dehydrogenase 4 (SCD4), acetyl-CoA carboxylase (ACC), and fatty-acid synthase (FAS), which promote lipogenesis by favoring the production of fatty acids and triglyceride accumulation in the liver. This protein further inhibits retinoid X receptor alpha (RXR-α) and PPAR-α, which are transcription factors involved in the regulation of mitochondrial carnitine palmitoyl-transferase type 1 (CPT-1), a mitochondrial enzyme which catalyzes the transportation of fatty acids into the mitochondria for β-oxidation [57,58,59,60]. As a result, CPT-1 is downregulated, which leads to mitochondrial dysfunction and activation of lipid oxidation in peroxisomes and the ER. The resulting end products of peroxidation; 4-hydroxynonenal (4-HNE) and malondialdehyde (MDA), exacerbate the oxidative stress, leading to steatosis [61].

The second proposed mechanism involves the strong association between hepatic steatosis and IR [62]. In hepatic IR, the clearance of glucose in the liver is impaired and is later compensated for by an increase in insulin production by the pancreas; however, this leads to not only hyperinsulinemia but also an overstimulation of lipogenesis, ultimately resulting in steatosis [63]. IR may result from the downregulation of insulin receptor substrate signaling 1 (IRS-1) due to an excess of free fatty acids (FFAs), tumor necrosis factor alpha (TNF-α), or suppressor of cytokine signaling 3 (SOCS3) [64], where the HCV core protein is once again implicated in the enhancement of FFA absorption [65]. This inhibits glucose uptake by glucose transporter type 4 (GLUT-4), thereby increasing both blood glucose and insulin levels, leading to steatosis [66]. Although this exact mechanism in HCV patients remains elusive, the few ideas that have been proposed suggest an interaction between the HCV core and NS5A protein and other elements involved in regulating lipid metabolism.

## 4. HCV-Related Steatosis and Extrahepatic Manifestations

HCV has been implicated in multiple extrahepatic disorders including metabolic disturbances such as MetS, atherosclerosis, diabetes mellitus, and IR [67,68,69]. CHC is commonly associated with steatosis, which is in turn linked to MetS, a host-related factor known to significantly accelerate progression of liver fibrosis in CHC [70,71,72,73]. MetS affects approximately 33% of the population in the developed world, where 33% of MetS patients develop NASH [74]. IR is believed to be the key pathogenic factor between NAFLD/NASH and MetS; evidently, the links between CHC and MetS [75,76], NAFLD and MetS [77], and NAFLD and CHC have been established. NAFLD and CHC are associated with an increased prevalence of CVD and T2D [78]. This is not surprising considering that MetS is an established risk factor for CVD and T2D [79,80,81]. In confirming the above-mentioned associations, Leornado et al. demonstrated that fat accumulation and liver fibrosis might be common determinants for the development of T2DM and CVD in patients with NAFLD, HCV, or HIV [78].

**Figure 2:**
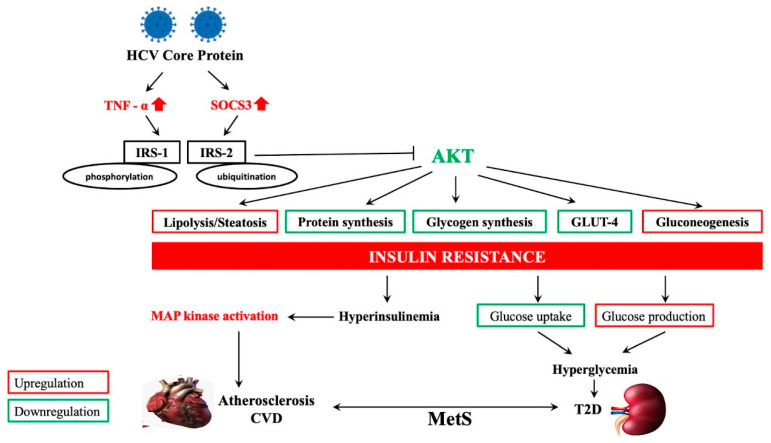
Extrahepatic disorders associated with hepatitis C virus. Red and green illustrate upregulation and downregulation, respectively. HCV: hepatitis C virus; TNF-α: tumor necrosis factor alpha; SOCS3: suppressor of cytokine signaling 3; IRS-1: insulin receptor substrate signaling 1; IRS-2: insulin receptor substrate signaling 2; AKT: protein kinase B or Akt signaling pathway; MAPK: mitogen-activated protein kinase signaling pathway; CVD: cardiovascular disease; MetS: metabolic syndrome; T2D: type 2 diabetes.

Similar complications in NAFLD patients have been confirmed by further studies [11,82,83,84,85]. The high CVD risk in NAFLD patients with MetS could be due to an increase in fibrosis stage, steatosis grade, or oxidative stress [86], which are collectively induced by FFAs. HCV infection has similarly been identified as a potential risk factor for both T2DM- and CVD-related complications [87,88]. HCV core protein upregulates TNF-α and SOCS3, causing the phosphorylation and ubiquitination of IRS-1/IRS-2, respectively, preventing it from associating with the insulin receptor and further blocking the activation of AKT. Since AKT has the responsibility of regulating numerous metabolic functions including lipolysis, protein and glycogen synthesis, gluconeogenesis, and GLUT-4, the result is IR [89]. Finally, IR may result in hyperglycemia in T2DM patients, as well as CVD, through the activation of the intracellular mitogen-activated protein kinase (MAPK) signaling pathway (involved in pathogenesis of cardiac and vascular disease) [90]. This mechanism is illustrated in Figure 2.

The risk of CVD is further increased in CHC through chronic vascular inflammation [91], and more timely screening of patients with hepatic steatosis for various extrahepatic manifestations could significantly help identify those at high risk and improve liver disease outcomes in HCV patients. It is imperative for clinicians to identify both high-risk patients and extrahepatic manifestations of steatosis earlier in the disease course to improve liver disease outcomes.

## 5. Molecular Mechanisms of HCV Genotype-Specific Steatogenesis

The effect of steatosis on CHC progression appears to be genotype-specific, where steatosis is mostly associated with host factors (obesity, diabetes, hypertension, and MetS) in G-1/2 [23] and viral proteins in G-3 infection [24,39]. Considering the analogy between HCV steatosis and NAFLD patterns, HCV-related steatogenesis may occur through the following steps: increased lipogenic substrates, increased de novo lipogenesis, decreased oxidation of fatty substrates, and decreased export of hepatic fatty substrates into the blood stream. Moreover, these NAFLD-like mechanisms of steatogenesis apply to all HCV genotypes with a few proposed differences where G-3 CHC seemingly amplifies steatogenic molecular mechanisms associated with NAFLD through significant changes in MTP, PPAR-α, SREBP-1c, and phosphate and tensin homolog (PTEN) [92]. Contrary to G-1/2, G-3 CHC patients exhibit significantly reduced MTP and PPAR-α activity, which downregulates exportation of lipogenesis and β-oxidation, respectively. There is also a possibility that G-3 CHC activates SREBP-1c more efficiently than G-1 through inactivation of PTEN, leading to de novo lipogenesis [92,93,94,95]. These G-3 CHC-magnifying steatogenic mechanisms might be due to specific differences in the core protein amino-acid sequence in this genotype [96]. On the other hand, the pathogenic mechanisms behind the “metabolic type” of HCV-associated steatosis remain elusive. However, recent studies have implied that IR, obesity, and dysregulation of adipocytokines may be among the factors involved [97,98].

**Figure 3:**
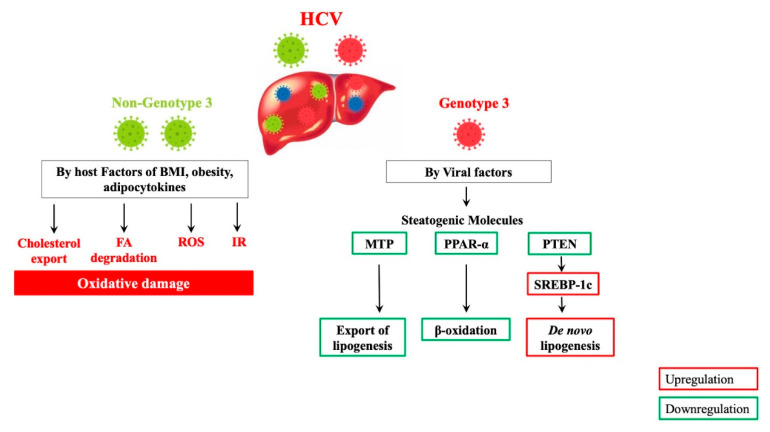
Hepatitis C virus genotype-specific molecular mechanisms of steatogenesis. Red and green illustrate upregulation and downregulation, respectively. HCV: hepatitis C virus; BMI: body mass index; FA: fatty acid; ROS: reactive oxygen species; IR: insulin resistance; MTP: microsomal triglyceride transfer protein; PPAR-α: peroxisome proliferator-activated receptor alpha; β-oxidation: beta-oxidation; PTEN: phosphate and tensin homolog; SREBP-1c: sterol regulatory element-binding protein 1c.

Additionally, the effect of oxidative damage on the histological and metabolic features of CHC are more evident in non-G-3, whereby CHC-infected patients in this group experience more severe steatosis. As a result, IR and oxidative stress are considered independent risk factors for steatosis in this group [99]. Furthermore, gene expression analysis from a study revealed how certain steatogenic pathways involving increased fatty-acid degradation and decreased cholesterol export are significantly induced in G-1 as opposed to G-3 livers [95]. These genotype-specific steatogenic pathways associated with HCV are illustrated in Figure 3.

Indeed, steatosis was observed in 87% of G-3 and 56% of G-1 HCV patients where the carbohydrate-responsive element-binding protein (ChREBP), a regulator of lipid metabolism, was found to be significantly expressed in G-1-infected livers. The precise role of ChREBP in lipid homeostasis remains controversial where, an overexpression of this protein maintained insulin signaling sensitivity and induced expression of the fatty-acid-regulating acetyl-CoA carboxylase enzyme while at the same time, knocking down this gene improved hepatic steatosis and IR in obese mice [95,100].

## 6. The Impact of Antiviral Therapy on HCV-Related Steatosis, Extrahepatic Manifestations, and HCC

Steatosis may predict treatment failure in CHC patients; however, this relationship is less well understood, [31,101]. The IFN-based therapy era was challenged with applying changes in lipid metabolism and liver steatosis to successful HCV eradication; as a result, studies have suggested that hepatic steatosis negatively impacts SVR following treatment [102,103]. Toyoda et al. suggested that steatosis and hepatic expression of genes involved in innate immunity are among factors associated with resistance to combination antiviral therapy with Pegylated interferon (PEG-IFN) and ribavirin [104]. The effect that steatosis has on HCV replication and interferon alpha (IFN-α) antiviral response was investigated in an infected cell culture model where a possible mechanism was proposed. In this study, intracellular fat accumulation following treatment with FFAs reduced the phosphorylation of signal transducer and activator of transcription 1 and 2 (Stat1 and Stat2)-dependent interferon beta (IFN-β) promoter activation, thereby hindering response to IFN-α and viral clearance. Furthermore, FFA treatment induced endoplasmic reticulum (ER) stress response and downregulated the interferon alpha receptor subunit 1 (IFNAR1) of the type I IFN receptor, which ultimately impaired JAK/STAT signaling, as well as antiviral response [105].

**Figure 4:**
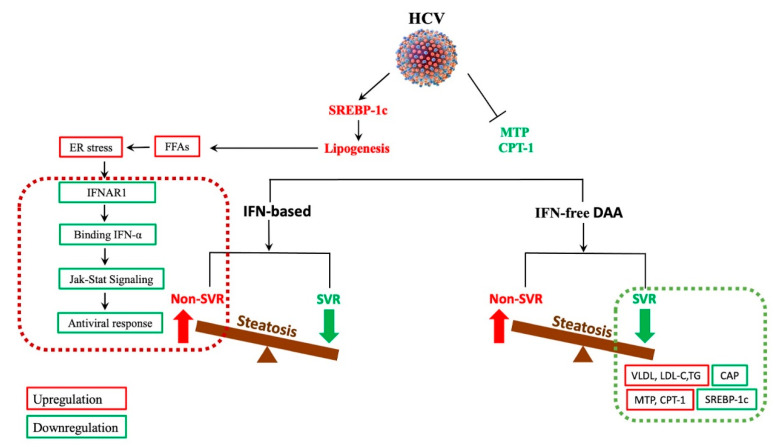
The effect of antiviral therapy on hepatitis C virus-associated steatosis. Red and green illustrate upregulation and downregulation, respectively. HCV: hepatitis C virus; SREBP-1c: sterol regulatory element-binding protein 1c; MTP: microsomal triglyceride transfer protein; CPT-1: carnitine palmitoyltransferase-1; FFAs: free fatty acids; ER: endoplasmic reticulum; IFNAR1: interferon alpha and beta receptor subunit 1; IFN-α: interferon-alpha; JAK/STAT: Janus kinase and signal transducer and activator of transcription signaling; IFN-based: interferon-based; SVR: sustained virological response; IFN-free DAA: interferon-free direct-acting antivirals; VLDL: very-low-density lipoprotein; LDL-C: low-density lipoprotein cholesterol; TG: triglycerides; CAP: controlled attenuation parameter.

IFN-free DAAs, which target the viral replicative machinery, have since replaced IFN-based therapies in HCV treatment, having successfully cured a significant number of patients, including those at high risk of HCC or with associated conditions such as renal dysfunction, CVD, and MetS [106,107]. Even though DAAs have significantly increased the treatment efficacy in HCV-infected patients [108,109], little is available about the mechanism behind the effect of hepatic steatosis on SVR following this therapy. What has been proposed, however, is the effect that DAAs and SVR have on HCV steatosis. Numerous studies have indicated that HCV infection upregulates SREBP-1c, [110], while downregulating MTP and CPT-1 [111], two elements which promote lipogenesis, as well as secretion of VLDL-C, and regulation of mitochondrial β-oxidation [60,112]. Therefore, according to Kawagishi et al., HCV eradication by DAA therapy should successfully downregulate SREBP 1c while upregulating MTP and CPT-1. As a result, lipogenesis is decreased while VLDL secretion is increased [113]. This article further demonstrated how HCV eradication by DAAs influences liver steatosis and atherogenic risk, where a decrease in CAP (a marker of steatosis) was suggested following SVR with this IFN-free therapy. Results showed that the overall changes in CAP were significantly elevated at SVR24; moreover, these changes were negatively correlated with baseline values of steatosis, whereby, in patients with severe steatosis at baseline, CAP values were decreased while those with lower baseline of steatosis experienced elevation of CAP. Since the impairment of VLDL secretion as a result of downregulation of MTP has been known to cause hypocholesterolemia and decreased triglyceride levels in HCV patients [114], successful treatment with DAAs might lead to elevation of LDL-C and triglycerides [115,116]. A summary of the response to IFN-based and IFN-free DAAs in HCV-associated steatosis is illustrated in Figure 4.

Similarly, in another study, patients with liver steatosis experienced elevated LDL-C and triglyceride levels accompanied with elevated sdLDL-C (small dense low-density lipoprotein cholesterol) at SVR24 [113]. In contrast, LDL-C levels in patients with non-SVR, remained unchanged following DAA treatment. Indeed, a positive correlation between sdLDL-C and NAFLD has been previously reported in patients diagnosed with NAFLD, suggesting the prospect of sdLDL level as a new biomarker of NAFLD [117], and possibly an even better predictor of CVD than LDL-C [118,119]. Hashimoto et al. further reported that total cholesterol levels following an SVR with DAA treatment were significantly increased in the ledipasvir/sofosbuvir group (87.45 to 122.5 mg/dL; *p* < 10^−10^) than in the daclatasvir and asunaprevir group (80.15 to 87.8 mg/dL; *p* = 0.0056) [116]. Whether or not this increase in serum LDL-C concentrations post DAA treatment relates to the specific combination of the DAA therapy or a decline in HCV core protein remains unclear.

The impact of steatosis on HCV genotypes has been investigated in several studies with varying results. While several studies have suggested no connection between steatosis and SVR in genotype [31,120], others have insisted that a lack of steatosis could significantly predict SVR in these patients [121]. Initially, G-3 HCV infection was considered the most challenging to treat during the IFN era [22]; however, this concept seemed to differ in patients with steatosis, where a significant reduction in steatosis after a SVR from IFN-based treatment was observed in patients with G-3 HCV, along with no change in either G-1- or G-3-infected patients without a SVR [24]. Similarly, in another study including 1428 naïve patients, the presence of steatosis after IFN therapy was associated with a lower SVR (*p* < 0.001), while steatosis was significantly reduced in G-3 HCV patients who achieved a SVR [31]. Regarding the varying presentation of liver steatosis and virological response to therapies among G-1 and 3, Meissner et al. suggested that, since steatosis in G-1 patients is not primarily mediated by HCV (which is the case for G-3), achieving a SVR regardless of the type of therapy might not solve the already increased fibrosis progression perpetuated by metabolic factors [115].

There are reports where a significant elevation in CAP levels following DAAs was reported to be genotype-specific [26,28]. In the latter reports, this elevation was observed in G-1/2, suggesting that this particular group may potentially be more susceptible to a decreased response to both IFN-based and IFN-free DAA treatment regimens. Other studies have also hypothesized that the differences between G-1/2 and G-3 in their response to DAA therapy could be due to transcriptional alterations in pathways involved in both lipid and inflammation metabolisms [95]. In this study, they further discovered a significant difference following treatment with DAAs among the genotypes in the altered liver gene expression, where 2151 genes were differentially expressed with a >1.5-fold difference between them.

A successful outcome following DAAs was reported in a retrospective clinical study where liver biopsies of patients before and after DAAs were examined to measure steatosis and fibrosis. DAA treatment managed to decrease steatosis and hepatic inflammation in most patients, except for a few with bridging fibrosis before treatment who either suffered persistent lymphocytic portal inflammation, decompensated cirrhosis, and HCC or developed cholangiocarcinoma post treatment. Consequently, HCV-infected patients who either have advanced fibrosis at treatment initiation or steatosis should be closely monitored for liver-related complications. Underlying NAFLD has, therefore, been associated with increased incidence of HCC in CHC patients following a SVR by DAAs [52]. A reduction in liver steatosis was also reported by Kobayashi et al. in a study involving 57 patients with CHC who achieved a SVR following DAA treatment [122]. In this study, the assessment of liver stiffness and steatosis based on transient elastography (TE) and CAP revealed a significant increase in total cholesterol and LDL-C levels, as well as a decrease in CAP levels from baseline to SVR48, particularly in steatosis-positive patients. As a result, it was suggested that liver steatosis is reduced in patients with a SVR following DAA therapy.

In addition to steatosis improvement, DAAs have been associated with a reduction in IR, thereby significantly decreasing the occurrence of two major extra-hepatic manifestations of CHC: T2DM and CVD. While investigating the effect of SVR on severity of carotid atherosclerosis in HCV patients undergoing DAA treatment, Petta et al. reported a decrease in the carotid intimal–medial thickening, 9–12 months post SVR [123]. Similarly, HCV clearance among 2204 HCV-infected patients was independently associated with a reduction in CVD (OR, 4.716; 95% CI: 1.832–12.138; *p* = 0.001) [124]. Interestingly, Di Minno et al. suggested a link between SVR and improvement in endothelial function where the flow-mediated dilation (FMD), a cardiovascular (CV) risk marker, was improved 12 weeks after DAA treatment [125]. While numerous studies have indeed confirmed an improvement in CV events due to DAA-induced SVR [126,127,128], others argue that DAAs may exert a cardiotoxic effect particularly in HCV-infected patients whose left-ventricular function is impaired [129]. Chen et al. further emphasized that HCV eradication by DAAs may negatively impact lipid metabolism, thereby significantly increasing LDL-C and central arterial stiffness, which are significant predictors of hypertension and CVD [130]. On another note, virus eradication with DAA regimens is linked to an improvement in parameters of glucose metabolism and IR [131,132]. As a result, a decrease in diabetes rates has been reported in HCV patients following DAA treatment [128]. The direct mechanisms via which HCV reverses the altered glucose and lipid homeostasis remain unsolved; however, HCV eradication was shown to be a major contributor in numerous studies.

Indeed, achieving a SVR with DAAs has proven to be a significant turning point in CHC infections; however, unforeseen increases in HCC recurrence and/or occurrence rate among HCV patients treated with DAA combination have led to the efficacy of this regimen being questioned [133,134]. Another study specifically proposed that early HCC occurrence may be correlated to the use of sofobusvir (SOF)-based therapy without ribavirin (RBV) [135], emphasizing the protective role of RBV on HCC onset [136]. According to Kobayashi et al., this may be partly explained by RBV’s inhibitory effect on regulatory T (Treg) cells, thereby assisting HCV-specific CD8^+^ T cells in eliminating HCV-infected hepatocytes [137].

In summary, while it has been proposed that hepatic steatosis negatively impacts SVR following IFN-based treatment through defective JAK/STAT signaling, better IFN-free DAAs have significantly improved steatosis. Since significant changes in steatosis have been particularly linked to G-3 HCV-infected patients who achieved a SVR, further understanding the underlying molecular pathways between different HCV genotypes might improve the treatment success of both IFN-based and IFN-free DAAs. An assessment on whether or not interventions specifically aimed at reducing the degree of steatosis should be considered as a means to improve antiviral efficacy. In addition, a SVR following DAAs significantly reduces the risks of CVD and T2DM (by improving endothelial function and glucose metabolism or IR), as well as HCC, particularly with the use of ribavirin. Patients undergoing SOF treatment without RBV require monitoring.

One of the limitations of this review is that despite having shed light on the effect of DAAs on HCV steatosis, the impact that HCV steatosis has on the efficacy of antiviral therapies remains a mystery. Moreover, the exact molecular mechanisms underlying the latter event, as well as those behind DAA restoration of the altered glucose and lipid metabolism, remain unsolved. In order to monitor steatosis, as well as maximize the efficacy of DAA therapy in CHC patients, these issues need to be addressed.

On the other hand, this review highlighted that, in as much as HCV steatosis and metabolic abnormalities are acknowledged risk factors for accelerated fibrogenesis, impaired treatment response to IFN therapy, and development of HCC, their clinical association with the improved DAAs demands further investigation. We were able to provide an up-to-date overview on the effect of DAAs and SVR on HCV steatosis among different genotypes, as well as propose significant changes in steatosis markers which could be useful therapeutic targets towards improving the efficacy of antivirals. This review went further to suggest that transcriptional alterations in pathways that are genotype specific could very well explain these changes, an aspect of this field that we believe is under-investigated. Lastly, interventions aimed at reducing the degree of steatosis in HCV-infected patients as a means to improve treatment efficacy have been neglected; therefore, this review hopes to present an opportunity to explore that aspect in future studies.

## 7. Conclusions

This review illustrates a significant relationship between CHC infection and hepatic steatosis through a combination of both viral and metabolic factors, further revealing a significant association with both hepatic and extrahepatic manifestations. The presence of NAFLD/NASH, otherwise known as MAFLD, has proven to be a significant marker of progressive liver disease and virologic response to HCV treatment, where the severity and frequency is significantly greater in G-3, while G-1 is associated with a poor response to both IFN-based and IFN-free DAA therapy. The severe consequences resulting from steatosis presence in CHC including increased risk of hepatic fibrosis and/or HCC, as well as decreased virologic response to antiviral therapy, all stress the urgency of investigating the complex derangements in host insulin and lipid metabolism. This will be extremely useful in devising specific therapies for HCV-related steatosis in CHC infection, particularly in G-3 patients.

## Figures and Tables

**Table 1:** HCV-related steatosis increases hepatocellular carcinoma risk. HCV: hepatitis C virus; IFN: interferon; HCC: hepatocellular carcinoma; SVR: sustained virologic response; BMI: body mass index; AFP: alpha-fetoprotein; DAAs: direct-acting antivirals; PR: Pegylated interferon plus ribavirin; ND: not determined.

Study	Number of Patients	Antiviral Therapy	Antiviral Response		HCC Occurrence/Recurrence (%)	HCC-Related Risk Factors
			SVR	Non-SVR		
Kurosaki et al., 2010 [47]	1279	IFN	393	886	68/1279~5%	High-grade steatosis, advanced fibrosis, non-SVR, older age, male sex, high BMI
Tanaka et al., 2007 [48]	266	IFN	266	0	6/266~2.6%	Steatosis, older age, fibrosis
Ohata et al., 2003 [49]	161	IFN (71/161)	20	51	71/71~100%	Steatosis, aging, cirrhosis, no IFN treatment
Takuma et al., 2007 [50]	88	Curative resection	ND	ND	55/88~63%	Steatosis, fibrosis stage, surgical procedure outcome, number and size of tumor
Pekow et al., 2007 [51]	94	Liver transplantation	ND	ND	32/94~34%	Steatosis, older age, AFP
Ji, D., et al., 2021 [52]	1735	IFN-free DAAs and PR	1336	399	54/1336~4.4%	NAFLD, older age, higher AFP level, higher liver stiffness measurement, diabetes mellitus

## Data Availability

Exclude this statement.
